# 

**Published:** 1886-06

**Authors:** 


JUNE. 1886.

### THE AMERICAN JOURNAL OF DENTAL SCIENCE.

LIBRARY
INDEXED
S. G. O.

PUBLISHED MONTHLY.

$2.50 PER ANNUM.

EDITED BY

F. J. S. GORGAS, M. D., D. D. S.

VOL. 20 THIRD SERIES. No. 2.

BALTIMORE:
SNOWDEN & COWMAN.

LONDON:
TRUBNER & CO., 60 PATERNOSTER ROW.

